# Contrasting patterns of genetic population structure in tropical freshwater eels of genus *Anguilla* in the Indo-Pacific

**DOI:** 10.1016/j.heliyon.2021.e07097

**Published:** 2021-05-20

**Authors:** Takaomi Arai, Hussein Taha

**Affiliations:** Environmental and Life Sciences Programme, Faculty of Science, Universiti Brunei Darussalam, Jalan Tungku Link, Gadong, BE 1410, Brunei Darussalam

**Keywords:** *Anguilla*, COI, Dispersal, Migration, Population, Tropical anguillid eels

## Abstract

Freshwater eels, genus *Anguilla*, have a distinctive catadromous life history, which could be associated with certain oceanic current systems and offshore spawning sites. Thus, migration and dispersion patterns are believed to be important factors influencing the population structure of each species. Temperate eel species are well studied, while little research has been conducted on the tropical counterparts that comprise two-thirds of all eel species. The population structure of three tropical species, *A. marmorata*, *A. bicolor bicolor* and *A. bengalensis bengalensis*, which are distributed widely in the Indo-Pacific region, were explored by means of DNA sequence analysis of mitochondrial cytochrome c oxidase subunit I (COI). This study suggests that *A. bicolor bicolor* might have two genetically distinct populations (fixation index, F_ST_ = 0.891; p < 0.001) that co-occur geographically in the Indo-Pacific region, while *A. marmorata* and *A. bengalensis bengalensis* might have a panmictic-population structure in this region. This study is the first to explore the population genetic structure of *A. bengalensis bengalensis*. The present results also suggest plausible dispersion and migration of these tropical species into their continental habitats.

## 1 Introduction

The freshwater eels of the genus *Anguilla* Schrank, 1798 consists of 19 species and subspecies [1, 2, 3]. Freshwater eels are categorised into either temperate or tropical species based on their geographical habitats [4]. The temperate and tropical eels consist of six and 13 species/subspecies, respectively. Their distribution ranges are almost worldwide except for the continental habitats in the eastern Pacific and South Atlantic oceans [1]. These freshwater eels have a unique catadromous life history with various ranges of oceanic migration from less than 100 to more than 5000 km [5]. The growth habitats and/or spawning sites of each eel species partly or completely overlap with at least one other eel species. The fundamental biology and ecology of temperate eels are generally well understood in term of life history, but those of tropical eels, which make up two-thirds of all freshwater eel species are very rudimentary [5].

Each species of freshwater eel has been hypothesized to have its own specific migration loop, and subsequently, any temporal and/or spatial changes in the migration loop could lead to speciation [6]. This hypothesis could in principle explain the divergence between sympatric eel species such as the Atlantic eels. The spawning sites of the two Atlantic eels, *A. anguilla* and *A. rostrata* overlap in the Sargasso Sea [7, 8]. However, the differences in their life history characteristics, such as lengths of larval duration, recruitment periods and peaks of spawning seasons, diverge and maintain their migration loops [9, 10, 11], although the existence of natural hybrids in the Atlantic eels has been found in the North Atlantic [12, 13]. Similarly, the spawning sites of a temperate eel, *A. japonica*, and a tropical eel, *A. marmorata*, also overlap in the western North Pacific Ocean. However, these eels are allopatrically distributed in Southeast and East Asian countries due to the different optimal temperatures required upon recruitment to continental habitats [14]. These findings indicate that both biotic and abiotic factors influence migration loops and divergence in freshwater eels. However, the divergence mechanisms in most of the eel species are still unclear.

The Indo-Pacific region is a suitable location to study the divergence of freshwater eels because there are multiple species, including *A. marmorata*, *A. bicolor* and *A. bengalensis*, which are sympatrically distributed in this region [1]. *Anguilla marmorata* is the most widespread species [1, 15] and is distributed from the east coast of Africa to the Indo-Pacific Ocean [5]. *Anguilla marmorata* has also been discovered in the Caroline Islands [16], the Galapagos Islands [17] and the Palmyra Atoll [18], indicating that its distribution range is much farther east than previously thought. *A. bicolor* is the second most widespread species and is distributed from the eastern coast of Africa to the Pacific Ocean [1]. The distribution range of *A. bengalensis* is from the east coast of southern Africa to the west coast of northwest Sumatra of Indonesia and Peninsular Malaysia [1, 3, 19] and is believed to have the third widest geographical distribution among freshwater eels. Previous research on the life history characteristics has found that these three species have similar features, such as comparatively shorter larval stages, faster larval growth and longer spawning seasons than temperate species [19, 20, 21, 22, 23, 24, 25].

A multiple-population structure was previously observed in *A. marmorata* from the Indian and Pacific oceans [26, 27, 28, 29]. *Anguilla bicolor* has been differentiated into two subspecies: *A. bicolor bicolor* in the Indian Ocean and *A. bicolor pacifica* in the western Pacific Ocean [1]. There was no substantial difference observed between the samples obtained from the western and eastern sides of the Indian Ocean by means of morphological and DNA analyses [30, 31, 32]. However, two mitochondrial sublineages of *A. bicolor bicolor* were instead found sympatrically in the Indian Ocean although this was not well supported by microsatellite markers [31, 32]. The population genetic structure of *A. bengalensis* has never been examined, although a study based on morphological observation (total number of vertebrae) found that *A. bengalensis* has two subspecies, *A. bengalensis labiata* and *A. bengalensis bengalensis*, in the western and eastern Indian Oceans, respectively [1, 33]. The genetic population structure of freshwater eels could provide clues to their biogeography and dispersion mechanisms in the ocean. Furthermore, research on the population structure of freshwater eels is necessary for establishing their protection, conservation and management, as well as for understanding the taxonomy and evolution of diadromous fishes.

The aim of the present study was to explore the population structure of *A. marmorata*, *A. bicolor bicolor* and *A. bengalensis bengalensis* in the Indo-Pacific region by means of mitochondrial DNA sequence analyses. We also discuss the potential dispersion and migration mechanisms and the importance of their population structure in the region.

## 2 Materials and methods

### 2.1 Eel samples

**Figure 1:**
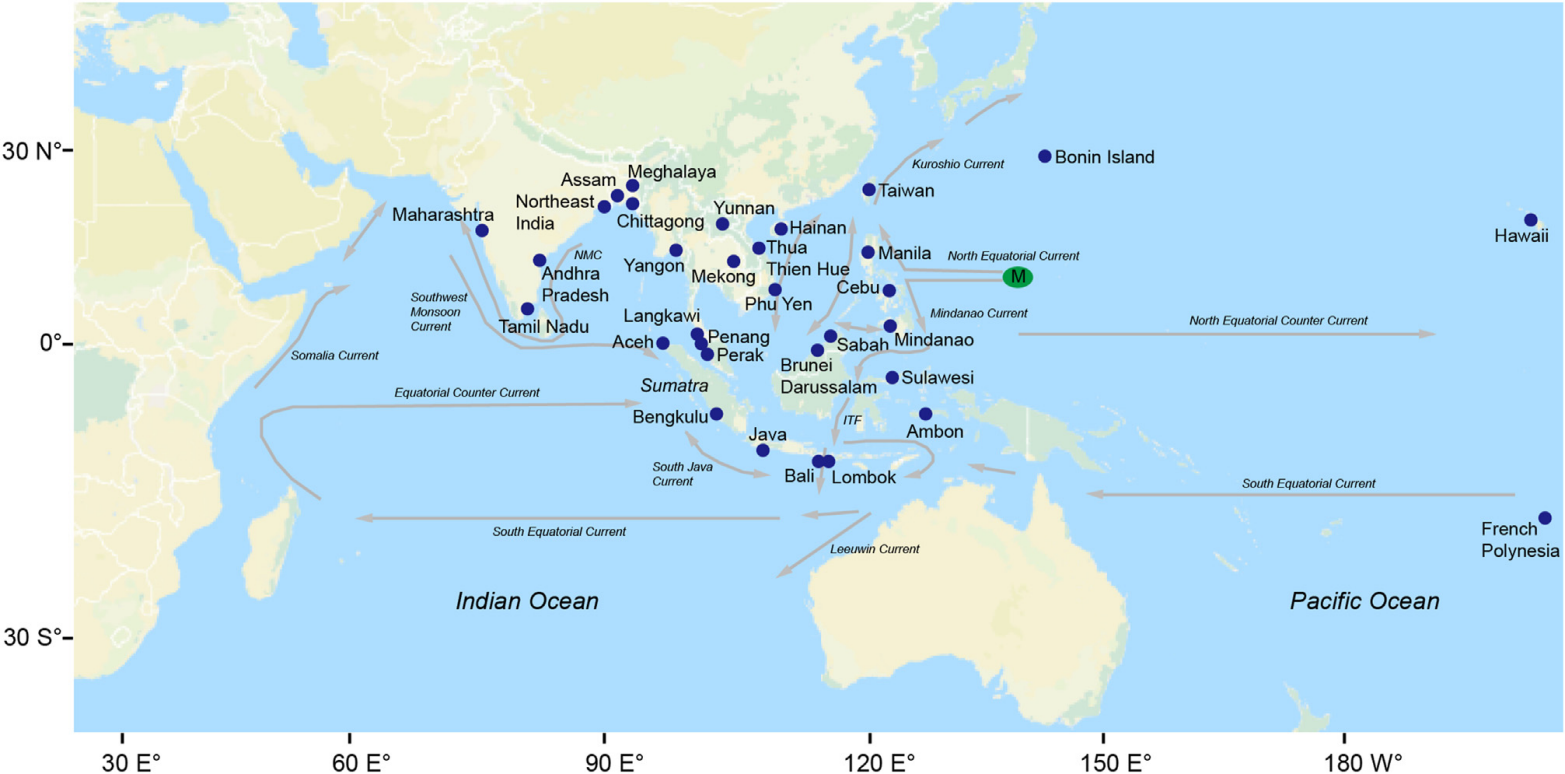
Map of locations of tropical freshwater eels, *A. marmorata*, *A. bicolor bicolor* and *A. bengalensis bengalensis* in the Indo-Pacific, which were used in our DNA analyses. The spawning site of *A. marmorata* in the North Pacific Ocean (green-highlighted M) and the oceanic currents (grey lines) in the Indo-Pacific are shown. The base map was downloaded from the OpenStreetMap (open access) at https://www.openstreetmap.org. ITF: Indonesian Throughflow, NMC: Northeast Monsoon Current.

A total of 511 specimens from 23 sites in the Indo-Pacific region from India to French Polynesia were used in the present study. From these 511 specimens, 35 specimens were collected from February 2015 to December 2019, and 476 specimens were obtained from the GenBank database (Tables 1, 2, and 3; Figure 1). In the 35 specimens, *A. marmorata* was collected in Sokong River, Lombok Island on 11 December 2019 (two specimens) and Dumoga River, Sulawesi Island on 2 August 2019 (21 specimens) in Indonesia, and *A. bengalensis bengalensis* was collected in Rusa River, Penang Island on 28 May 2015 (three specimens) and Perak River, Perak State from 2 April to 23 May 2015 (nine specimens) in Peninsular Malaysia, Malaysia. There were 164 specimens of *A. marmorata* originating from 6 sites in Indonesia, two sites in Vietnam and one site each in Brunei Darussalam, China, French Polynesia, Hawaii (USA), Japan, Malaysia, the Philippines, Taiwan and Thailand (Table 1, Figure 1). There were 262 specimens of *A. bicolor bicolor* originating from three sites in Indonesia, two sites in Malaysia, one site each from India and Myanmar, and six specimens of *A. bicolor pacifica* from two sites in the Philippines (Table 2, Figure 1). For *A. bengalensis bengalensis*, a total of 80 specimens originated from six sites in India, three sites each in Indonesia and Malaysia, and one site each in Bangladesh and China (Table 3, Figure 1). Our protocols followed the ethical guidelines for the use of animals of Universiti Brunei Darussalam (UBD) and were approved by the animal ethics committee at UBD.

**Table 1:** List of samples and haplotypes for phylogenetic and haplotype analyses in *Anguilla marmorata*.

Haplotype	No. of samples	Localities
H1	2	Indonesia: Bengkulu (MT155391, MT155392)
H2	65	French Polynesia (JQ431413); Indonesia: Aceh (KY618770, KY618775, KY618778, KY618787), Bengkulu (JQ665824, JQ665824, MT155393 - MT155446), Java (KU692248, KU692251, KU692252); Vietnam: Thua Thien Hue (MN067941)
H3	1	Indonesia: Bengkulu (MT155447)
H4	64	Indonesia: Ambon (AP007242), Bali (KU692249, KU692250), Lombok (1 sample from this study - LO4), Sulawesi (14 samples from this study - DI1, DI4, DI5, DI6, DI8, DI11, DI13, DI15, DI16, DI20, DI21, DI23, DI25, DI28); Brunei (MN315356); Japan: Bonin (MN315357); Malaysia: Sabah (MG324010 - MG324012); Philippines: Manila (KC970325, KC970327); Taiwan (KU942680, KU942730, KU942731); Thailand: Mekong (MG324009); Vietnam: Phu Yen (MK818584, MK818585), Thua Thien Hue (MN067927 - MN067932, MN067934, MN067935, MN067937, MN067939, MN067940, MN067942, MN067943, MN067946 - MN067951, MN067954 - MN067956, MN067958 - MN067960, MN067962 - MN067968, MN067970)
H5	2	Hawaii (DQ520999, DQ521000)
H6	1	Indonesia: Aceh (HM345929)
H7	1	China: Hainan (HQ141374)
H8	1	French Polynesia (JQ431414)
H9	1	Philippines: Manila (KC970326)
H10	1	Taiwan (KU885607)
H11	4	Indonesia: Sulawesi (2 samples from this study - DI22, DI30); Vietnam: Thua Thien Hue (MN067944, MN067969)
H12	1	Vietnam: Thua Thien Hue (MN067961)
H13	5	Indonesia: Sulawesi (1 sample from this study - DI19); Vietnam: Thua Thien Hue (MN067936, MN067945, MN067952, MN067957)
H14	1	Vietnam: Thua Thien Hue (MN067953)
H15	5	Indonesia: Sulawesi (1 sample from this study - DI26); Vietnam: Phu Yen (MK818583), Thua Thien Hue (MN067923, MN067925, MN067938)
H16	1	Vietnam: Thua Thien Hue (MN067933)
H17	2	Indonesia: Lombok (1 sample from this study - LO3); Vietnam: Thua Thien Hue (MN067926)
H18	1	Vietnam: Thua Thien Hue (MN067924)
H19	1	Brunei (MN315355)
H20	1	Japan: Bonin (MN315358)
H21	1	Indonesia: Sulawesi (1 sample from this study - DI7)
H22	1	Indonesia: Sulawesi (1 sample from this study - DI18)
H23	1	Indonesia: Sulawesi (1 sample from this study – DI27)

**Table 2:** List of samples and haplotypes for phylogenetic and haplotype analyses in *Anguilla bicolor*.

Species	Haplotype	No. of samples	Localities
*Anguilla bicolor pacifica*	H1	5	Philippines: Cebu (AP007237), Mindanao (MT107686 - MT107688, MT107690)
	H2	1	Philippines: Mindanao (MT107689)
*Anguilla bicolor bicolor*	H3	7	Myanmar: Yangon (AP007236); Indonesia: Bengkulu (MT155547 - MT155551), Java (MT155546),
	H4	61	Indonesia: Aceh (KY618776, KY618785 - KY618786), Bengkulu (MT155492 – MT155533), Java (KU692247, MT107683, MT107684, MT155534 – MT155542); Malaysia: Langkawi (KF182304), Penang (KM875505, MT107673, MT107675)
	H5	1	Malaysia: Penang (KM875503)
	H6	2	Indonesia: Aceh (KY618780); Malaysia: Penang (KM875504)
	H7	162	India: Andhra Pradesh (KP979655, MG675613); Indonesia: Aceh (KY618771, KY618779, KY618783, KY618788, KY618790, KY618792), Bengkulu (MT155552 - MT155658), Java (MT107677 - MT107682, MT155659 - MT155698); Malaysia: Penang (MT107676)
	H8	1	Indonesia: Aceh (KY618768)
	H9	1	Indonesia: Aceh (KY618769)
	H10	1	Indonesia: Aceh (KY618773)
	H11	1	Indonesia: Aceh (KY618777)
	H12	1	Indonesia: Aceh (KY618781)
	H13	1	Indonesia: Aceh (KY618782)
	H14	2	Indonesia: Aceh (KY618784); Malaysia: Penang (MT107674)
	H15	1	Indonesia: Aceh (KY618789)
	H16	4	Indonesia: Aceh (KY618791), Bengkulu (MT155545), Java (MT155543, MT155544),
	H17	1	Indonesia: Aceh (KY618793)
	H18	1	Indonesia: Aceh (KY618794)
	H19	1	Indonesia: Aceh (KY618795)
	H20	1	Indonesia: Java (MT107683)
	H21	6	Indonesia: Bengkulu (MT155484 – MT155489)
	H22	2	Indonesia: Bengkulu (MT155490), Java (MT155491)
	H23	3	Indonesia: Bengkulu (MT155699 - MT155701)

**Table 3:** List of samples and haplotypes for phylogenetic and haplotype analyses in *Anguilla bengalensis bengalensis*

Haplotype	No. of samples	Localities
H1	1	India: Maharashtra (JX260828)
H2	1	India: Maharashtra (JX887590)
H3	1	India: Maharashtra (JX887591)
H4	25	Bangladesh: Chittagong (MK572027, MK572028, MK572030, MK572031); China: Yunnan (KM610420); India: Andhra Pradesh (KR021973, KT895265, MG675618); Indonesia: Aceh (KY618774), Bengkulu (MT155471 - MT155483); Malaysia: Langkawi (KF182302), Penang (KM875500, KM875501)
H5	1	Malaysia: Langkawi (KF182303)
H6	37	Bangladesh: Chittagong (AP007246, MK572029); China: Yunnan (KM610417, KM610419); India: Assam (KP982886), Meghalaya (KX355465); Indonesia: Aceh (KY618767), Bengkulu (MT155448 - MT155468), Java (MT155469, MT155470); Malaysia: Penang (KM875498), Penang (2 samples from this study - SP28, SP29), Perak (KP897130, 3 samples from this study - HR7B, HR8B, HR10B)
H7	1	Malaysia: Penang (KM875499)
H8	1	Malaysia: Penang (KM875502)
H9	1	Malaysia: Penang (KT72853)
H10	1	Indonesia: Aceh (KY618772)
H11	2	China: Yunnan (KM610418); Malaysia: Perak (1 sample from this study - HR9B)
H12	1	India: Tamil Nadu (MG747436)
H13	1	India: Northeast India (MG736325)
H14	1	Malaysia: Perak (1 sample from this study - HR3B)
H15	1	Malaysia: Perak (1 sample from this study - HR4B)
H16	1	Malaysia: Perak (1 sample from this study - HR5B)
H17	1	Malaysia: Perak (1 sample from this study - HR6B)
H18	2	Malaysia: Penang (1 sample from this study - SP27), Perak (1 sample from this study - HR11B)

### 2.2 Mitochondrial DNA analysis

Genomic DNA samples were extracted using the QIAGEN DNeasy Blood & Tissue Kit according to the manufacturer's instructions. The mitochondrial cytochrome c oxidase subunit I (COI) was amplified by polymerase chain reaction (PCR) using QIAGEN 2x *Taq* PCR Master Mix according to the manufacturer's instructions. The PCR primers used were either universal primers [34] or primers that we designed for *Anguilla* eels, AngF (5′TCA CCC GTT GAT TCT TTT CT3′) and AngR (5′CCG ATA GCC ATT ATT GCT CAG3′). The PCR products were purified using a QIAGEN QIAquick Gel Extraction Kit. The purified COI fragments were sent to a sequencing service provider, where they were sequenced bi-directionally with the same primers.

MEGA X [35] was used to align, inspect and edit forward and reverse sequences. The resulting contig sequences were uploaded to the GenBank database with accession numbers MW375913–MW375947. To identify the species, a BLAST search in the GenBank database was used to match the contig sequences with the GenBank reference sequences. MEGA X was used to carry out multiple sequence alignment via ClustalW. The multiple alignment was trimmed at both ends to remove columns with missing data, resulting in lengths of 506 bp for *A. marmorata*, 445 bp for *A. bicolor*, and 529 bp for *A. bengalensis bengalensis*.

MEGA X was used to construct phylogenetic trees via neighbour joining (NJ), maximum parsimony (MP) and maximum likelihood (ML) algorithms. In this study, only the representative MP trees are shown. The NJ tree was constructed using the Kimura 2-parameter model. The MP tree was obtained using the Tree-Bisection-Reconnection method with search level 1, in which the initial tree was obtained by 1000 random addition replicates. The ML tree was constructed with the best-fit nucleotide substitution model, the Tamura 3-parameter (T92) for *A. marmorata* and *A. bengalensis bengalensis*, and T92 with a discrete gamma distribution (+G; 5 categories). A heuristic search starting with the initial tree (based on the NJ and BioNJ algorithms) was conducted using the nearest-neighbour-interchange method. For all trees, all codon positions were included, and a bootstrap test with 1000 replicates was also carried out. Quantification of DNA polymorphisms and statistical tests (Tajima's test and fixation index, F_ST_) were performed using DnaSP 6 [36]. A haplotype network was constructed via the median joining method using Network 10 (www.fluxus-engineering.com).

## 3 Results

From the 35 specimens that were amplified and sequenced in this study, the BLAST search showed that 23 and 12 specimens were identified as *A. marmorata* and *A. bengalensis bengalensis*, respectively, with high identity matches of 99–100%. Therefore, by molecular analysis, this study confirmed the occurrence of *A. marmorata* in Lombok and Sulawesi of Indonesia, and *A. bengalensis bengalensis* in Penang and Perak of Peninsular Malaysia. Haplotype analysis showed the presence of 23, 23 and 18 different haplotypes in *A. marmorata*, *A. bicolor* and *A. bengalensis bengalensis*, respectively (Tables 1, 2, and 3).

**Figure 2:**
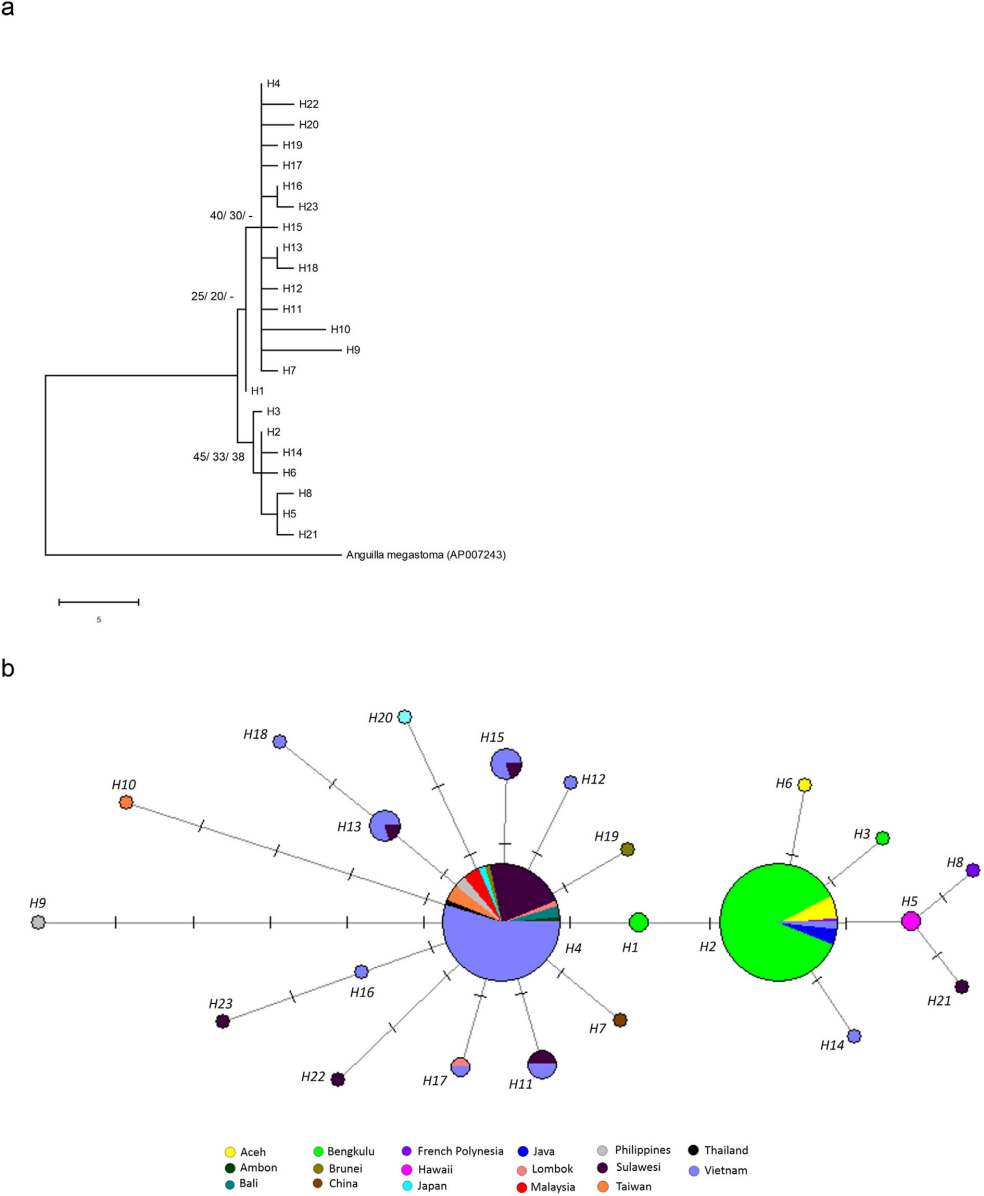
Phylogenetic tree and haplotype network of *Anguilla marmorata*. (a) Representative MP tree with the consistency, retention and composite indices of 0.60, 0.69 and 0.60, respectively. The bootstrap percentages for MP/NJ/ML are shown. (b) Haplotype network showing sample sites represented by different colours with the size being proportional to the sample size and the mutational steps symbolised by dashes.

For *A. marmorata*, both the MP and NJ trees seemed to show two clades or groups, but they were not supported by the bootstrap probabilities (Figure 2). The ML tree also did not show the occurrence of these two groups. This suggests that *A. marmorata* from the Indo-Pacific region formed a panmictic population. Similarly, the haplotype network (Figure 2) for *A. marmorata* also suggests a panmictic population structure, as no distinct grouping could be observed, some haplotypes, especially H4, were shared between several localities. It is interesting to note that for H2, this haplotype was shared by Indonesia (Aceh and Bengkulu on Sumatra Island, and Java Island), French Polynesia and Vietnam, which were of considerably distant from each other geographically.

**Figure 3:**
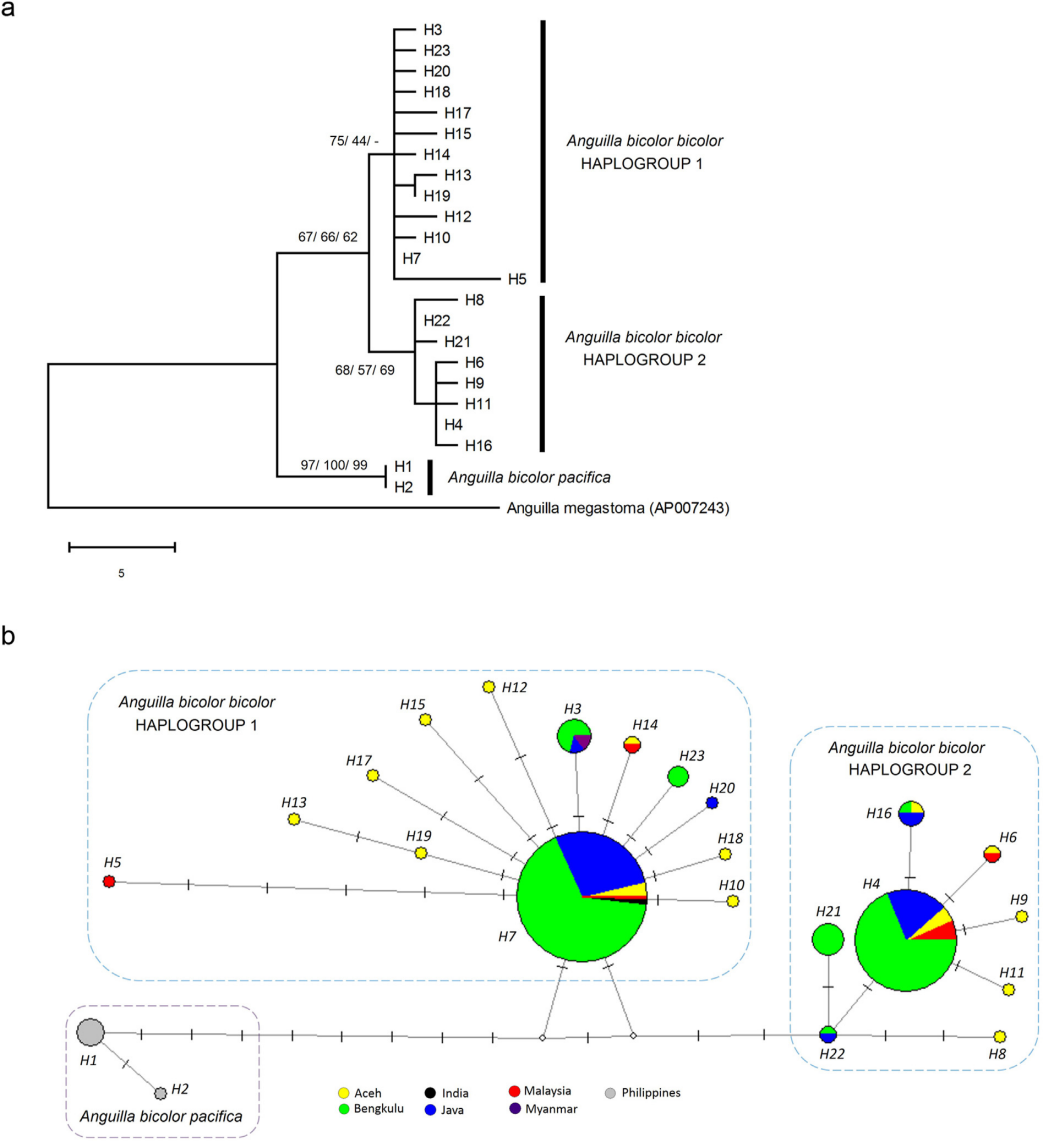
Phylogenetic tree and haplotype network in *Anguilla bicolor*. (a) Representative MP tree with the consistency, retention and composite indices of 0.70, 0.81 and 0.71, respectively. The bootstrap percentages for MP/NJ/ML are shown. (b) Haplotype network showing sample sites represented by different colours with the size being proportional to the sample size and the mutational steps symbolised by dashes.

For *A. bicolor*, both phylogenetic trees and haplotype networks supported the divergence of this species into two subspecies, *A. bicolor bicolor* and *A. bicolor pacifica* (Figure 3). This is supported by the high bootstrap probabilities observed in the MP, NJ and ML trees. For *A. bicolor bicolor*, two haplogroups or populations could be clearly observed in the MP and NJ trees, with higher bootstrap probabilities shown by the MP tree. However, the ML tree did not support the occurrence of these two haplogroups. Two haplogroups of *A. bicolor bicolor* were also clearly observed in the haplotype network (Figure 3). Both groups could be found together in the same localities – Aceh and Bengkulu on Sumatra Island, Java Island and Peninsular Malaysia.

**Figure 4:**
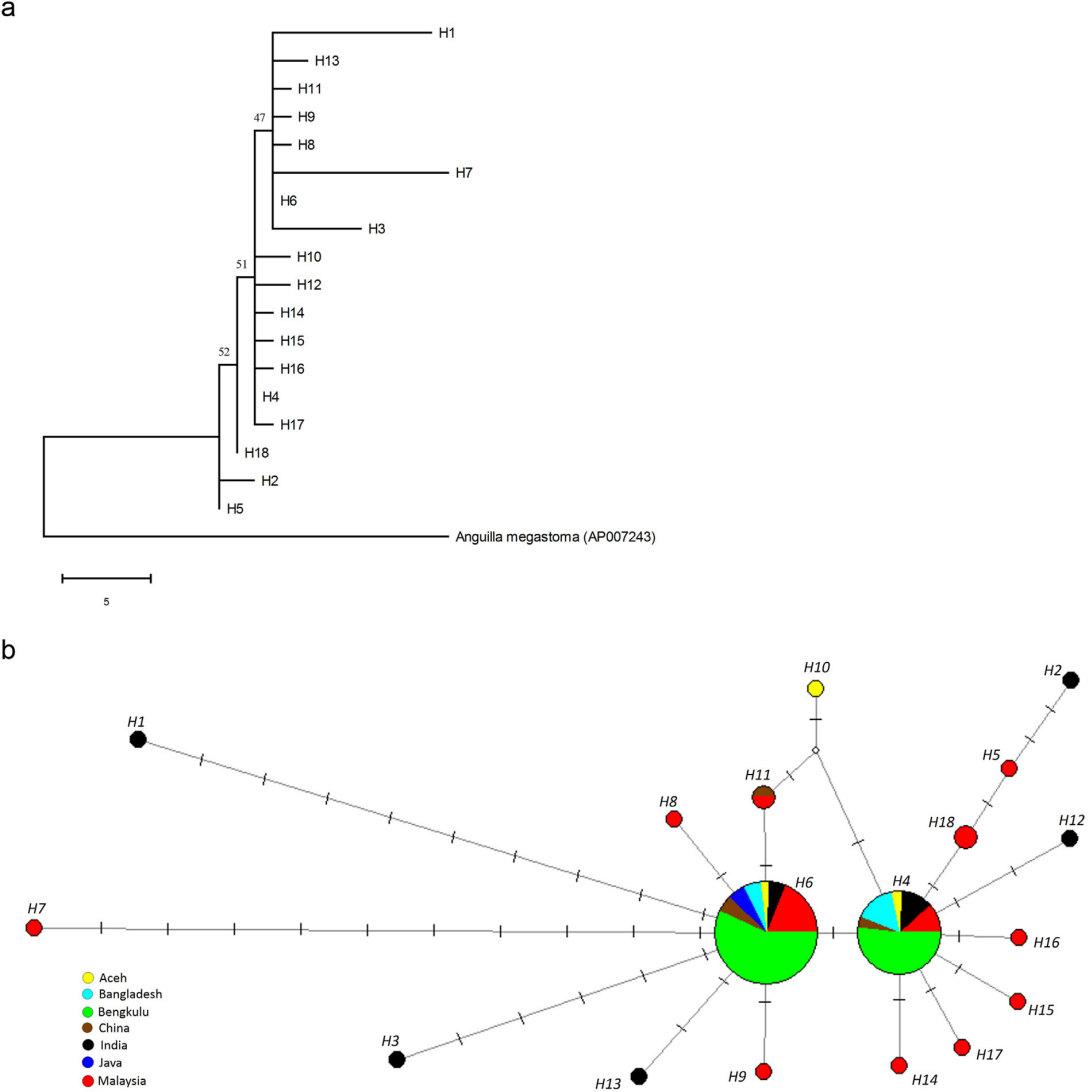
Phylogenetic tree and haplotype network in *Anguilla bengalensis bengalensis*. (a) Representative MP tree with the consistency, retention and composite indices of 0.67, 0.75 and 0.71, respectively. The bootstrap percentages for MP/NJ/ML are shown. (b) Haplotype network showing sample sites represented by different colours with the size being proportional to the sample size and the mutational steps symbolised by dashes.

For *A. bengalensis bengalensis*, no distinct grouping could be observed in the MP tree (Figure 4). Similarly, neither NJ nor MP showed any well-supported grouping. This suggests that this species formed a panmictic population in the Indo-Pacific region. Similarly, the haplotype network for *A. bengalensis bengalensis* also did not show any distinct grouping. Furthermore, two haplotypes, H4 and H6, were shared by several localities. Interestingly, three haplotypes, H1, H3 and H7, were more divergent than the other haplotypes.

As shown in Table 4, the panmitic populations of *A. marmorata* and *A. bengalensis bengalensis* were found to have higher haplotype diversity and nucleotide diversity than the two populations of *A. bicolor bicolor*. The haplogroup 2 of *A. bicolor bicolor* had slightly higher genetic variability than the haplogroup 1. However, when all samples were pooled together, *A. bicolor bicolor* showed higher average no. of nucleotide differences and nucleotide diversity compared to the other two species, indicating the samples were more divergent. Tajima's test (Table 4) showed negative Tajima's D values in all populations, which suggests recent population expansion, although the D statistic was not significant for one of the *A. bicolor bicolor* populations. The F_ST_ value between the haplogroup 1 and haplogroup 2 of *A. bicolor bicolor* was calculated to be 0.891 (p < 0.001), suggesting the two populations were genetically different. The F_ST_ value between *A. bicolor bicolor* and *A. bicolor pacifica* was calculated to be 0.887 (p < 0.001).

**Table 4:** DNA polymorphism and Tajima's test.

	Am	Abc	Abc1	Abc2	Abg
No. of sequences	164	261	183	78	80
No. of polymorphic sites	30	28	19	8	41
No. of haplotypes	23	21	13	8	18
Haplotype diversity, Hd	0.691	0.560	0.215	0.383	0.694
Average no. of nucleotide differences, k	1.548	1.982	0.314	0.602	1.642
Nucleotide diversity, π	0.00306	0.00445	0.00071	0.00135	0.00310
Tajima's D	-2.04 (p < 0.05)	-1.55 (ns)	-2.42 (p < 0.01)	-1.59 (ns)	-2.57 (p < 0.001)

Am: *Anguilla marmorata*; Abc: *Anguilla bicolor bicolor* (all samples); Abc1: *Anguilla bicolor bicolor* (haplogroup 1); Abc2: *Anguilla bicolor bicolor* (haplogroup 2); Abg: *Anguilla bengalensis bengalensis*.

ns: not significant.

## 4 Discussion

The present study explored the population structure of three tropical freshwater eels, *A. marmorata*, *A. bicolor bicolor* and *A. bengalensis bengalensis*, since there are only a limited number of studies that had examined their population structure. The study showed that there were two genetic populations of *A. bicolor bicolor* present in the Indo-Pacific region (Figure 3), whereas *A. marmorata* and *A. bengalensis bengalensis* each showed a panmictic population structure (Figures 2 and 4). Given that *A. bicolor bicolor* can be found on both sides of the Indian Ocean, it could be hypothesized that its population structure should be similar to that of *A. marmorata*. However, this was not the case. The panmictic population of *A. marmorata* is also in disagreement with the results from previous studies, whereby *A. marmorata* was found to have a multiple-population structure [26, 27, 28]. Although there were inconsistencies between these previous studies, Ishikawa et al. [26] and Minegishi et al. [27] proposed five populations – North Pacific Ocean, South Pacific Ocean, western Indian Ocean, eastern Indian Ocean (Sumatra Island) and Guam populations – while Gagnaire et al. [28] supported the existence of three divergent populations only – North Pacific Ocean, South Pacific Ocean and western Indian Ocean populations. The present study used mitochondrial COI sequences, but the previous studies examined different DNA markers (mitochondrial control region, mitochondrial cytochrome *b*, microsatellites, AFLP), which might explain the different results observed. In comparison with the mitochondrial markers used in those studies, the COI sequences analysed in this study had much lower number of polymorphic sites. Different sampling localities might also explain the different results observed. In addition, the present study also did not analyse any *A. marmorata* specimens from the western Indian Ocean due to no mitochondrial COI sequences are available in the GenBank database and no *A. marmorata* specimens are available in this study. In this study, *A. marmorata* from Aceh and Bengkulu on Sumatra Island did not form a distinct eastern Indian Ocean population, and *A. marmorata* from French Polynesia and Hawaii did not form a South Pacific population, as in the previous studies [26, 27, 28]. However, this study found that *A. marmorata* from these localities formed a panmictic population with the specimens from other localities, especially Java, Sulawesi and Vietnam (Figure 2).

As *A. marmorata* was previously found to have two genetic populations in the Indian Ocean (western and eastern Indian Ocean populations), these populations were suggested to originate from different spawning areas [26, 28]. Two plausible spawning sites of *A. marmorata* could exist in the Indian Ocean, i.e., in the southwestern Indian Ocean and off Sumatra Island [29]. If the latter is true, *A. marmorata* should be dominantly recruited to nearby regions. However, on the Java and Sumatra islands, *A. bicolor bicolor* was found to be the dominant species, accounting for 85% of eels found in these regions [37]; thus, these regions are not part of the major distribution zone for *A. marmorata*. Similarly, in northwestern Peninsular Malaysia, which is adjacent to Sumatra Island, *A. bicolor bicolor* was found to be the most dominant species, accounting for 88.1% of the eels found in this region, whereas *A. bengalensis bengalensis* was the second-most dominant species at 11.7%, while *A. marmorata* was present at only 0.2% [38]. These findings suggest that the transportation of the leptocephali of *A. marmorata* to the Sumatra and Java islands was likely not from this spawning site in the Indian Ocean but was possibly from the Pacific Ocean. *Anguilla marmorata* is also distributed in Brunei Darussalam [39] and Sabah of Malaysia [40, 41] on Borneo Island, and on Bali and Lombok islands of Indonesia, which are near the Sumatra and Java islands (Figure 1). Oceanic currents flow from the North Pacific and South Pacific oceans through off eastern Borneo Island (Makassar Strait) and off northern Australia to the Sumatra and Java islands [42] (Figure 1). Therefore, *A. marmorata* on the Sumatra and Java islands might be transported from spawning site(s) in the Pacific Ocean.

One spawning site of *A. marmorata* in the Pacific Ocean is located in the western North Pacific [43] (Figure 1). Brunei Darussalam, Sabah (Malaysia), Thailand, and Vietnam are located in the South China Sea (Figure 1). The South China Sea is subjected to seasonal monsoons; during the northwest monsoon, the surface circulation is directed southward, while during the southeast monsoon, the circulation is in the opposite direction [42]. The larvae of *A. marmorata* might be transported (i) from the northern South China Sea (between the northern Philippines and southern Taiwan) by the North Equatorial Current (NEC) and Kuroshio Current, and/or (ii) from the southern South China Sea through the Celebes (Sulawesi) and Sulu Seas by the NEC and the Mindanao Current (Figure 1). *A. marmorata* from Ambon, Bali and Lombok could be further transported by the Indonesian Throughflow via the Makassar Strait (Figure 1). This one spawning site would ensure a homogenous population of *A. marmorata* in the Indo-Pacific region. An area on the northwest of Fiji in the South Pacific Ocean is also thought to be a spawning area of *A. marmorata* [44]. Nevertheless, *A. marmorata* from different potential spawning sites in the Pacific Ocean could still form a panmictic population through secondary contact.

The early life history parameters of *A. marmorata* were almost the same in the Pacific region, with the average durations of the leptocephalus stage ranging between 110 days (Philippines) to 128 days (Indonesia) and the ages at recruitment ranging from 145 days (Japan, Philippines and Taiwan) to 155 days (Indonesia) [20, 21, 24, 45, 46]. These parameters are slightly higher than those of *A. marmorata* recruited to the southwestern Indian Ocean with the average duration of the leptocephalus stage of 97–105 days and the age at recruitment of 120–128 days [23, 47]. The difference in the early life history parameters between the Pacific Ocean and the Indian Ocean suggest that there might be distinct populations across the Indo-Pacific region.

*Anguilla bicolor* from the Indian Ocean (*A. bicolor bicolor*) and the Pacific Ocean (*A. bicolor pacifica*) were found to diverge from each other (Figure 3), and the outcomes of our COI gene sequence analysis were similar to those of previous studies using microsatellites, mitochondrial control region and cytochrome *b* sequences [31, 32]. Furthermore, the previous studies also found that on the western and eastern sides of the Indian Ocean, *A. bicolor bicolor* was observed to have two divergent mitochondrial sublineages [31, 32]. These two sublineages or populations co-occur geographically in the Indian Ocean. The present results also supported the occurrence of two divergent haplogroups or populations of *A. bicolor bicolor* in the eastern Indian Ocean but not in the western Indian Ocean because no GenBank sequences nor specimens are available to be analysed in this study. However, based on the previous studies [31, 32], it is speculated that the western Indian Ocean might similarly have these two divergent haplogroups or populations. These different *A. bicolor bicolor* haplogroups or populations in the eastern Indian Ocean could occur together within the same habitats on Java Island, Sumatra Island (Aceh and Bengkulu) and Peninsular Malaysia (Figure 3). Spawning sites of *A. bicolor bicolor* are thought to be located off west Sumatra Island in the eastern Indian Ocean [37] and off east Madagascar Island in the western Indian Ocean [47, 48]. However, no strong evidence is yet available to support these proposed spawning sites of *A. bicolor bicolor*, unlike the spawning site of *A. marmorata* in the North Pacific Ocean where its eggs, smaller leptocephali and fully matured adults have been found. These two geographically different spawning areas of *A. bicolor bicolor* might explain the occurrence of the two divergent haplogroups or populations in the eastern Indian Ocean. Furthermore, the ages of recruitment of this species between the eastern (148–202 days in Java, Indonesia) and western coasts (68–96 days in Mascarene Islands) of the Indian Ocean were substantially different from each other [20, 47], which suggest the occurrence of different spawning sites.

*Anguilla bicolor bicolor* in the eastern Indian Ocean has a long duration of the leptocephalus stage (larval age) (119–171 days) [20], and the spawning season is year-round [19, 22]. These life history strategies might enable *A. bicolor bicolor* to disperse widely across the Indian Ocean. Seasonal changes in the South Equatorial Current and the long larval migration period in the Indian Ocean, which was estimated to be more than one year in anguillid eels [49], might be capable of mixing *A. bicolor bicolor* larvae from different spawning sites. It is speculated that the two divergent mitochondrial haplogroups or populations observed in this study originated from two different spawning sites, presumably in the eastern and western Indian Ocean, which were later mixed together during larval migration. However, it is also possible for these two haplogroups to originate from the same spawning site or gene pool and the observed divergence between the two haplogroups could be due to other factor or mechanism. Moreover, based on limited microsatellite markers, previous studies did not support the two mitochondrial sublineages of *A. bicolor bicolor* [31, 32]. This led to an alternative hypothesis that there could be an incomplete lineage sorting or an ancestral population that split into two groups which later made a secondary contact [31]. Further studies are therefore needed to resolve the population genetic structure with more sampling and nuclear markers.

In contrast to *A. bicolor bicolor*, *A. bengalensis bengalensis* had a panmictic population in the Indian Ocean (Figure 4). This could be because the species has a narrower distribution range than that of *A. bicolor bicolor* in the Indian Ocean. The distribution range of *A. bengalensis bengalensis* includes India, Sri Lanka, Myanmar, Andamans, the west coasts of North Sumatra of Indonesia and Peninsular Malaysia [1, 33, 38, 50, 51, 52, 53, 54, 55], and the species is restricted to the eastern half of the Indian Ocean only, unlike *A. bicolor bicolor*, which is distributed widely throughout the ocean. Although the present study suggests a panmictic population in *A. bengalensis bengalensis*, several haplotypes (H1, H3 and H7) were observed to show divergence from the other haplotypes (Figure 4). Therefore, the occurrence of sub-populations in the Indian Ocean might be possible. Information on the life history of *A. bengalensis bengalensis* [19, 38, 54, 55] is scarce compared with those of *A. marmorata* [14, 20, 21, 23, 24, 25, 44, 45, 46, 47, 56, 57] and *A. bicolor bicolor* [19, 20, 21, 22, 38, 47, 54, 55, 58, 59, 60], and only a few leptocephali have been collected from off Sumatra Island and in the eastern Indian Ocean [37, 61]. Therefore, further intensive studies on the spawning site, life history and molecular genetic analyses are needed to understand the transportation and dispersion mechanisms of this eel.

## 5 Conclusions

This study has revealed the unique and different population structure in *A. marmorata*, *A. bicolor bicolor* and *A. bengalensis bengalensis*, which are sympatrically distributed in the Indo-Pacific region. *A. bicolor bicolor* and *A. bengalensis bengalensis* were proposed to be transported from spawning site(s) in the Indian Ocean. However, *A. marmorata* from the eastern Indian Ocean (Sumatra and Java islands) was likely not transported from the Indian Ocean but from spawning site(s) in the Pacific Ocean. The transportation and recruitment mechanisms are likely complicated and different among these three species in the Indo-Pacific region. The molecular evidence from this study calls for further research on the life history, stock assessment and protection of the populations of tropical freshwater eels in the region. To understand the dispersal and transportation mechanisms, further study is needed to investigate the spawning sites of all tropical species together with their population structure, phylogeny and life history analyses in the Indo-Pacific region.

## Declarations

### Author contribution statement

Takaomi Arai: Conceived and designed the experiments; Analyzed and interpreted the data; Contributed reagents, materials, analysis tools or data; Wrote the paper.

Hussein Taha: Performed the experiments; Analyzed and interpreted the data; Wrote the paper.

### Funding statement

This work was supported by 10.13039/100009100Universiti Brunei Darussalam under the Competitive Research Grant Scheme (UBD/OVACRI/CRGWG (003)) and under the Faculty/Institute/Centre Research Grant (UBD/RSCH/1.4/FICBF(b)/2020/029).

### Data availability statement

Data will be made available on request.

### Declaration of interests statement

The authors declare no conflict of interest.

### Additional information

No additional information is available for this paper.
